# Hospital and Institutional News

**Published:** 1919-07-12

**Authors:** 


### HOSPITAL AND INSTITUTIONAL NEWS.

### THE RED CROSS AMBULANCES.

FURTHER details now come to hand of the Home Service Scheme under the British Red Cross Society and the Order of St. John. Ambulances returned from war service are being divided between various county districts in England, Wales, and Ireland. There will be organised stations about thirty miles apart by road and under the control of the county directors. At the moment we understand that out of the ninety-nine county areas, eighty-nine have sent in applications for these vehicles, and that 207 ambulances have been allotted to seventy-six counties, nearly half of them being already delivered. Certain areas, such as the Isle of Wight, Guernsey, etc., do not require this assistance owing to peculiarity of local circumstances; and, as we recently pointed out in appealing for more ambulances in the densely populated areas, London, Birmingham, Bristol, and Middlesex will not be served until the needs of the more rural districts have been supplied. The value of this county distribution cannot be over-estimated, and we believe that every available Red Cross vehicle could be so disposed of and still leave a margin of need in outlying districts, but those responsible must not forget the frequent and urgent ambulance requirements in the towns, which have been particularly noticeable of late with the tendency for people, in England at least, to crowd more and more into the urban districts. The principle of this scheme is excellent, and it is hoped that it will be carried out to the end with a due appreciation of shortage proportional to population as well as to geographical distribution.

### TREATMENT OF EX-SOLDIERS.

WE would draw attention to an Army Council Instruction which states that, except in cases of urgency, discharged soldiers applying for treatment at a military hospital should, in the first instance, be referred to the War Pensions Committee, who will determine whether the man is eligible for care at such an institution. Military units cannot treat discharged men unless application is made authoritatively by the Ministry of Pensions or its branches. To some the measure may sound out of tune and sympathy with our feeling towards men who have perhaps given much for their country, but nevertheless, the procedure is obviously part of correct demobilisation measures and the correct working of pensions schemes. There must, in the first place, be definite division between the periods during which treatment, allowance and pension are paid, and a large number of further reasons might be added. The complication, slight though it in reality is, may easily give rise to a sense of grievance in certain instances and individuals, and in expectation of expressions of such we may remark that this measure is in reality essential among the many end-of-war changes.

### ACCOMMODATION FOR SICK SOLDIERS.

AN Order has been issued by the Local Government Board ere it was merged in the Ministry of Health, under Section 80 of the Public Health (London) Act, 1891, prescribing regulations and restrictions under which persons, not being poor persons chargeable to the rates, suffering from trench fever, malaria, or dysentery may be received into hospitals provided by the Metropolitan Asylums Board. This Order provides that cases in which it is applicable shall be received into the hospitals of the Board only when there is room available beyond the requirement of those diseases occurring among poor persons chargeable to the rates and subject to exceptional cases provided for in Article 11 of the Order. Such cases can only be admitted upon the recommendation of the Medical Officer of Health.

### ACCESSORY FACTORS IN FOOD.

A MOST valuable and interesting memorandum has been drawn up by the committee appointed jointly by the Medical Research Committee and the Lister Institute for the guidance of those engaged in administration of food relief in the many poverty and famine-stricken districts which unfortunately exist in the world at the present moment. A concise scientific introduction to food-factor problems is provided, followed by an outline of the best practical applications which can be made of our present knowledge. From these notes one may, in view of their great importance in every branch of health work, make a few brief extracts. (*a*) It is unlikely that any danger of beri-beri will arise among the famine-threatened districts of Eastern Europe as long as wholemeal flour from rye, wheat, barley, maize, or peas, beans and lentils are provided. Mere shortage of food does not cause beri-beri, and poverty itself ensures that whole grain is consumed for purposes of economy. (*b*) To prevent rickets, children must be supplied with the *right kind of fat*; (1) full-cream milk should be given to artificially fed infants when possible; failing that (2) full-cream dried milk, or (3) full-cream condensed milk. (2) is preferred to (3), and in the case of ignorant or careless mothers even to (1), in order to prevent spread of infection and intestinal disorders. With (2) and (3), however, extra antiscorbutic factor must be provided, even in the form of expressed juice from green vegetables if other sources of vitamine are not at hand. The value of cod-liver oil is to be remembered, and also the fact that the nursing mother must take in her own diet these essential food factors if her milk is to transmit them to the child. (*c*) This also applies to the antiscorbutic factor, which can be supplied by allowing wheat, etc., to germinate, and cooking for the minimum time. In absence of more refined fruit juices, raw swede or turnip will give much vitamine, but all *canned and dried* vegetables are practically useless for the prevention of scurvy.

### LONDON WATER SUPPLY.

THE annual report on examinations of the London waters during the year ending in March 1919 was recently published, and from its pages one learns of some important changes which the experience and events of war have brought into waterworks management. Except during the most trying phases of the past years, when great temporary strain, both natural and artificial, was experienced, the average quality and purity of London's water has been wonderfully maintained, and the new methods of purification which made possible this success will probably be adopted by the Board in future. Certain waters, such as the New River, Chelsea, and East London, gave the worst bacteriological results (the *B. coli* test) obtained for some time, but undoubtedly floods were largely responsible, and with regard to Chelsea the reports state that these works "were asked to do too much in relation to the filtration area." Many points appear in favour of chlorination of the water, and this process has been both continued and extended. It is definitely superior to storage methods, particularly in winter, and is found to be more economical—in some ways, at least. Also it clears the waters from algæ and similar growth with remarkable efficiency, a boon to be readily appreciated by water engineers. Reservoirs were repeatedly seeded in flood-time, but when chlorinated water was once more the source of supply the algæ died out with satisfactory rapidity. A well-known problem in water supply is thus apparently nearing solution, and upon this application, together with other general and technical improvements, those responsible for the water researches are to be heartily congratulated.

### WHAT BECOMES OF MEDICAL WOMEN?

THE annual report of the London (Royal Free Hospital) School of Medicine for Women is always an instructive publication, and not its least interesting feature is the list of former students of the School, stating the present address and appointment of each student. We notice that in this list there are 707 names of medical women who are mostly working at their profession all over the world, twenty-three only being noted as retired from practice; 218 of the former students have married since their student days, and several have married medical men. A large number of the medical women mentioned hold hospital and other public appointments, and forty-one of them are engaged in resident appointments at voluntary hospitals. The medical woman came into her own during the war, and, so far as home institutions were concerned, her chief usefulness was to replace a medical brother on active service. Many women, of course, also served with the forces, and we notice a respectable list of former students who have been granted British and French honours, or who have been mentioned in despatches.

### A TOO PREVIOUS PRESS: VERIFY YOUR FACTS.

OUR *confrères* in the lay Press may welcome the correction that the Lord Chamberlain's office was charged with the duty of distributing tickets for the thanksgiving service at St. Paul's Cathedral last Sunday. In this respect the announcement made in the Press was erroneous. It has caused a great deal of trouble to the public and the officials. We mention the matter in the hope that before announcements of the kind affecting arrangements for the Peace celebrations are made they may be authenticated in every instance before publication.

### METHODS OF ADVERTISING.

FEW columns of the newspapers are now free from advertisements, and the letters from institutions which appear among the correspondence are the legitimate reward of the regular advertiser. It is something of a novelty, however, for a long letter to appear with the abbreviation (advt.) at its close. For this means that the letter has been directly paid for, and is not distinguished by type or position from the ordinary correspondence. Such, however, has been the fate of the letter issued by Sir William Treloar on behalf of the Cripples' Home at Alton, which appeared in the *Daily Telgraph* on June 30. We cannot help thinking that the newspaper and the institution would have been alike better served if instead of publishing the letter in this fashion an advertisement had separately appeared on the same day. The objection does not lie against the transaction itself, but against a method which deprives the letter of its chief value, and irritates the reader at finding an advertisement out of its place among the news. For it cannot be denied that the public takes more interest in correspondence than in most other parts of a general newspaper, and that the only effective way to prevent a letter from being read is to place the warning "advt." at the foot of it.

### LOSS UPON LOSS.

DEATH has been very busy amongst the leading supporters of King Edward VII.'s Hospital, Cardiff. The loss of Colonel Bruce Vaughan is the heaviest blow the institution has received during the past twenty years. And, in addition, during the past few months the hospital has been deprived by death of Mr. H. Woolcott Thompson, a trustee and most generous benefactor; Mr. George Birt, a zealous member of its committee; and, within the past few days, Mrs. Richard Cory, who in union with her children had recently provided the beautiful "Richard Cory" Ward as a memorial to her husband. On Saturday, the 5th inst., the chairman of the Finance Committee, Mr. John Morgan, died—an able and devoted friend of the hospital, held in the widest esteem and respect, and one whose influence was always exercised for the true welfare of the hospital and its patients. The cup is filled to overflowing, and now is the time for the younger generation to show its practical sympathy with the great hospital where both brains and money are wanted to carry on its fine traditions of usefulness.

### PEACE AND ST. THOMAS'S.

Now that St. Thomas's Hospital is badly in want of money, and that the yield from legacies last year was very slender, it is well that the public should remember that even last year practically half the beds were reserved for civilians. On the other hand, a big work has been undertaken for the treatment of war pensioners and disabled men, and that the latter may be thorough, as well as extensive, Sir Robert Jones has been placed in charge of the orthopædic department. To begin the peace in financial low water is a poor return for the services which St. Thomas's has rendered during the war. There has been no occasion in its history when a rally of its friends was better deserved.

### ST. BARTHOLOMEW'S LANDS.

VARIOUS freehold agricultural properties are, by the instructions of the Governors of St. Bartholomew's Hospital, to be offered for sale this month, the first sale being at Winchester House on July 17. The property includes 780 acres of land with two farms near Stevenage and Hitchin, and two more farms at Hatfield Broad Oak, Essex, to the extent of 345 acres. On July 22 a sale at Cambridge includes 1,280 acres at Hertford, and at a third sale on July 25, at Chelmsford, 3,150 acres in Essex are to be offered. This last area contains twelve farms. The object of the governors in disposing of real estate has not yet been expressed, but this form of property is selling very well at present, and there are plenty of good stock investments into which to put the proceeds. So the policy, on the face of it, appears to be quite sound.

### WAR HOSPITAL HISTORIES.

IT is noteworthy that war hospitals have been more articulate than traditional institutions. To the list of their histories we must now add the story of Hadfield Hospital, by Helen Macdonald. This, an Anglo-American hospital, situated at Wimereux, near Boulogne, was started on December 2, 1914, by Sir Robert and Lady Hadfield. It received over 3,000 British officers, 36 American officers, and 10,357 British non-commissioned officers and men. The value of its work is recorded in a letter from Lord Derby, who, in thanking Sir Robert, said: "You will, I hope, allow me to say that your generosity in maintaining the admirable Anglo-American Hospital at Wimereux has placed us under a great debt of gratitude. It was a source of pleasure to us, as it must have been to you, when Lady Hadfield's work was recognised in the last Honours Gazette." Lady Hadfield, who acted as lady superintendent, received the C.B.E. in 1917.

### A NEW USE FOR WAR HOSPITALS?

A SHORTAGE of cottage homes is complained of by Mr. F. Morris, the chairman of the Children's Country Holidays Fund, which gives to 20,000 London children a fortnight's holiday in the country. During the war, when the number of children benefited fell to this figure (it was over 40,000 in 1914), the chief difficulty was perhaps that of transport. Now few suitable cottage homes can be found. A request has been made to the British Red Cross Society to allow war hospitals on the verge of closing to be used to accommodate the children. We have every sympathy with this fund, because a fortnight in the country is of real use to a town child, whereas a day merely is of trifling value.

### WAR BONUSES AND SUPERANNUATION.

BERMONDSEY Board of Guardians have been called upon by all their employees to withdraw an agreement which they signed in 1915, under which they agreed that no war bonuses granted since then shall be taken into consideration in computing their superannuation allowances. The crisis was brought about when it was discovered that one of the subordinate officers was penalised to the extent of £14 a year through signing this agreement, and his fellows have asked that this agreement shall be reconsidered. At a meeting of the employees, at which some 400 were present, every branch of the services was represented, for the agreement affects the medical and nursing profession as well. Many of these war bonuses were granted when the wages of the ordinary workers were being automatically increased, and were given because the Guardians had to keep pace with the employers outside. Hence the argument that bonuses should be taken into account when the superannuation allowance is arranged. The Guardians have seen the injustice of the agreement, and are expected to instruct their clerk to withdraw it.

### THE INSTITUTIONAL POSTER.

WE have put in a word before now for the institutional poster, but it is, apparently, one of those things which must be seen to be believed in. Therefore we call attention to the coloured posters which were exhibited at the recent conference of the Children's Country Holidays Fund at the Guildhall. They are worth study, and should be seen at 18 Buckingham Street, Strand, the office of the fund. A good poster not only can be posted on a wall; it can be reproduced in the columns of newspapers in place of, or to supplement, the long written appeals which form the usual advertisements, at least in the provinces.

### THE FILM USED FOR TEACHING SURGICAL OPERATIONS.

THE Registrar of St. Lewis College of Physicians and Surgeons has an article in the current issue of the *Educational Film Magazine* dealing with the utilisation of films as a method of teaching the science of surgery. This method is enthusiastically recommended by Dr. Waldo Briggs, Dean of St. Lewis College, and Professor of Surgery at that institution. The spectator of a major operation sees but little, as the patient is frequently surrounded by assistants and the surgeons work fast. For a number of years Dr. Briggs has made many attempts to obtain films of operations, but it was not until the summer of 1918 that he came across a photographer who was able to work on a method which produced a satisfactory film. The means employed are as follows: A stage for the camera some 10 feet high was built overlooking the operating-table, and the photographer, with the aid of two powerful lights, was enabled to focus directly upon the surgeon's hand, following the knife into the incision, and thereby to photograph the entire operation from start to finish. In the course of Dr. Briggs' experiments it became plain that for the purposes of filming operations coloured people as patients were unsuitable. Although there was always plenty of light, the result obtained was only a dark shadow, through which nothing could be distinguished. On one occasion Dr. Briggs operated on a coloured man with a large lipoma tumour on the back of the neck. Although the picture was taken under exactly similar conditions as others on white people, absolutely nothing of the actual operation was distinguishable. The conclusion was come to that this was due to the shortness of the focus combined with the colour of the patient. Dr. Briggs is confident as to the future of the surgical film, and he considers that in but a few years any man turned out by a medical college using this method of instruction should be capable of performing any of the major operations.

### INVALID CHILDREN'S AID ASSOCIATION.

Under the auspices of the above body a conference was held at Manchester University on July 2 and 3. Papers were contributed by Doctor Kerr, of the Medical Research Committee, Doctor Rivers, Tuberculosis Officer, Barnsley District, Doctor Gertrude Hickling, and others. A general consensus of opinion was reached as to the desirability of promoting educational institutions on open-air lines, and of the provision of special accommodation for children suffering from surgical tuberculosis. With regard to the latter, it was mentioned that the military orthopædic hospitals, established so largely in this country during the past four years, might be turned over to children requiring orthopædic treatment. This would involve all crippled children, from whatever causes, being cared for by the one institution, a country branch to be provided for the tuberculous ones. We think, however, that Manchester, as yet without a hospital for tuberculous cripples, could not do better than follow the example of Sheffield, and the plans resolved on in the West of England, at Huddersfield, and in Essex, by keeping the accommodation for tuberculous children quite apart from that of other orthopædic cases. The treatment differs very largely in the two instances, because tuberculous patients require hygienic-dietetic measures, heliotherapy, and other special treatment. A visiting staff, too, is quite unnecessary for them, but a highly-trained resident medical officer is indispensable. This is not to say that anything that can be got out of the War Office should not be secured without delay, and, indeed, we believe that the estate at Alton, which Sir William Treloar and Dr. Gauvain have used so well, was acquired in this way after the South African War. The more one thinks of it, the more unsuitable does the plan above-mentioned appear. Non-tuberculous crippled children, who are mostly talipes or old infantile paralysis subjects, or those suffering from the sequelæ of rickets, are naturally delicate: they need all screening possible from infection, and should certainly be kept from the chance of meeting tuberculous children. There is, further, the question of possible friction with the public health authorities, who have chief charge of the latter disease and who, in the instances named, have made the best recommended provision for it—namely, institutions on the lines of Alton, of Bappenau, of Berck-sur-Mer.

### THE ASSETS OF A CHARITY.

The Chaplain of St. Bartholomew's Hospital, Rochester, the Rev. John Bailey, preached at Rochester Cathedral on Hospital Sunday. Speaking of the voluntary system and comparing it with the present craze for nationalisation, Mr. Bailey said that each represented an opposed tendency and that the least ill-ordered state is that in which the two aims are as far as possible reconciled. If under the Ministry of Health the medical profession becomes a branch of the civil service, and hospitals are placed under one great Government Department, he urged that the endowment of St. Bartholomew's Hospital shall be left sacred for the purpose to which the founder, Bishop Gundolph, devoted it. The poor, the sick are burdensome to themselves in being necessarily a burden to others. They are, in other words, liabilities, and the State, thinking only of its assets, can hardly be expected to care for them as liabilities. He might have added that the popular way to deal with liabilities is to "write them off," and this perhaps explains why official departments think more how to save their assets, their funds in hand, than of the needs to which they are supposed to minister. But St. Bartholomew's assets, in the eye of its founder, are the sick themselves! Here is the difference, and here the reason for the humanity of voluntary work and management.

### A DISCREDITED TRAFFIC.

Mr. E. C. Price, who is honorary Secretary of the Charity Voting Reform Association, gave some interesting facts about "voting charities" in a recent address in London. He remarked that for the past fourteen years no charity had been founded which made its benefits dependent on the votes of its subscribers. Thus the present generation regarded the voting charity as a curious survival. Fifty years ago, he added, unscrupulous people with money by trafficking in votes could secure a cheap annuity for a dependant. The only effective reform was total abolition. Mr. Price also said that a moderate estimate of the sum spent every year by unsuccessful candidates was £45,000. Unfortunately, the latter point is the sort of thing which makes more impression nowadays than the abomination of the voting system, the calculated cruelty and waste of which we have often pointed out. The system is, as we have said before, petty patronage in its last ditch, and its evils are only faintly relieved by the kindred system of "recommends," a noun deservedly unknown to the King's English.

